# Supplementary material for: Munc18 and Munc13 serve as a functional template to orchestrate neuronal SNARE complex assembly
Source: Nat Commun. 2019 Jan 8;10:69. doi: 10.1038/s41467-018-08028-6 (PMC6325239; doi:10.1038/s41467-018-08028-6)
Supplement: Supplementary file 1 — Supplementary information [file 41467_2018_8028_MOESM1_ESM.pdf]

Supplementary Information

Munc18 and Munc13 serve as a functional template to orchestrate neuronal SNARE complex assembly

Wang et al.

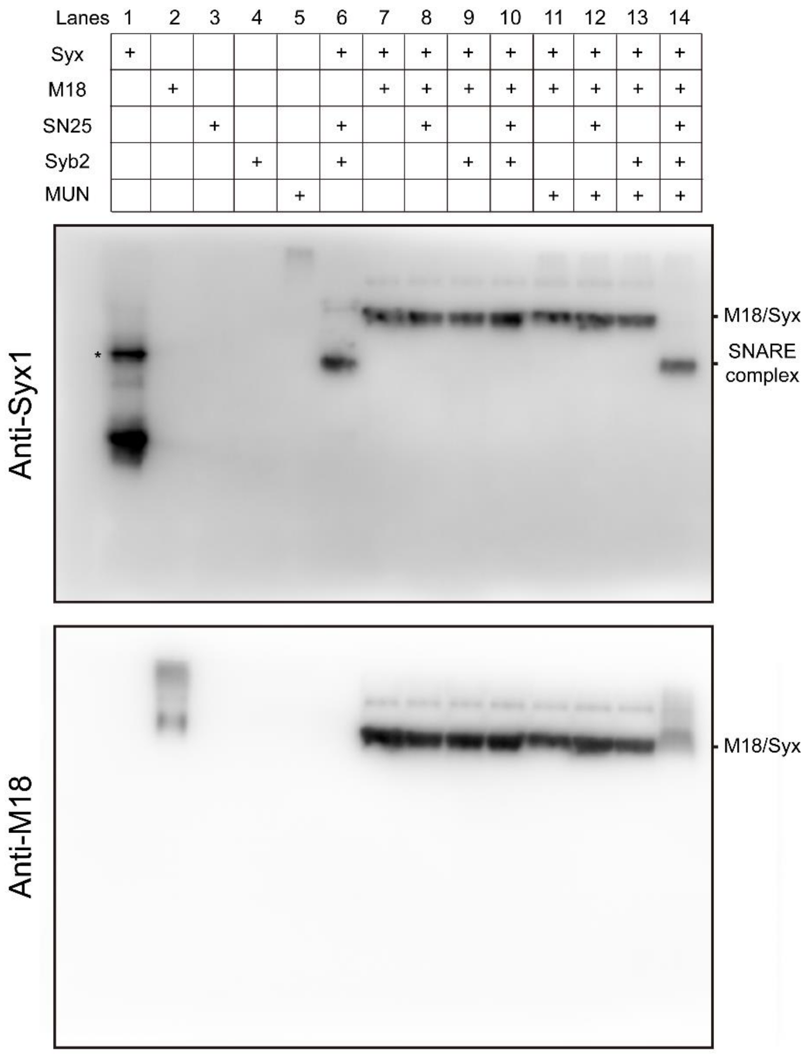

Supplementary Figure 1. Immunoblotting of the standard native-PAGE assay. Syx1 and Munc18-1 antibodies were applied as indicated, respectively. Syx1 bound to Munc18-1 exhibits a strong and clear band, while free Munc18-1 displays smeared band. The SNARE complex bands are as indicated. After the Munc18-1/Syx1 complex transits to the SNARE complex, Munc18-1 runs slightly faster than the isolated one (lane 14, lower panel), which suggests that there exists multiple interactions among Munc18-1, MUN, and the SNARE complex. Asterisk indicates potential aggregation of Syx1. Lane numbers are displayed at the top of the chart. In all conditions, Syx1 (residues 1–261) was 2 μM, Munc18-1 was 2 μM, SN25 was 10 μM, Syb2 (residues 29–96) was 10 μM, and MUN was 30 μM.

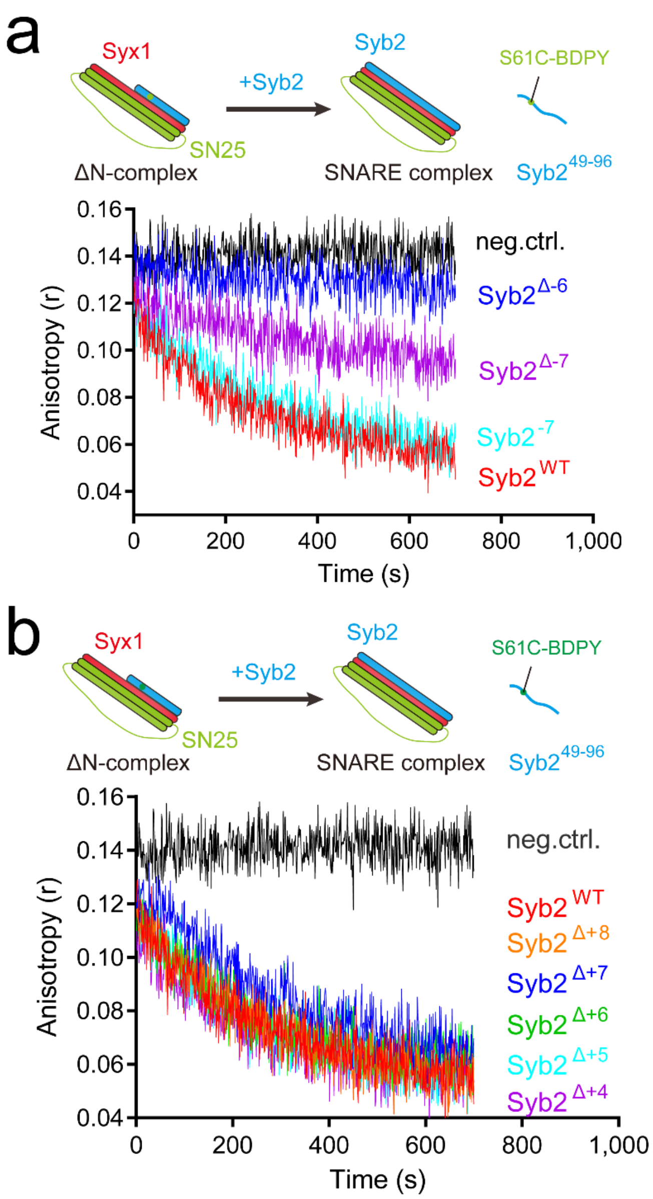

Supplementary Figure 2. SNARE N-terminal nucleation and zippering affected by the Syb2 N- and C-terminal truncations or mutations. Syb2$^{49–96}$ associates with SN25 and Syb2 to form a ΔN-complex (Syx1/SN25/Syb2$^{49–96}$), with its N-terminal SNARE binding layers being accessible for N-terminal nucleation and zippering with Syb2 (29–96); and subsequent N- to C-zippering of Syb2 (29–96) leads eventually to the disassociation of Syb2$^{49–96}$ from the ΔN-complex. As a consequence, the N-terminal nucleation/zippering ability of Syb2 (29–96) can be accordingly characterized by assessing the disassociation rate of fluorescence-labelled Syb2$^{49–96}$ with a time-based fluorescence anisotropy. **a** SNARE nucleation ability for the Syb2 N-terminal truncations/mutations analyzed by the Syb2$^{49–96}$-peptide displacement assay. **b** SNARE nucleation ability for the Syb2 C-terminal truncations analyzed by the Syb2$^{49–96}$-peptide displacement assay. Negative control represents pre-incubation of the N-terminal half of Syb2 (residues 29–55) in the reaction. Fluorescent dye (BOPY) was labeled at residue S61C of Syb2$^{49–96}$. In all experiments, the pre-assembled ΔN-SNARE complex (Syx1/SN25/Syb2$^{49–96}$) was 0.5 μM, and unlabeled Syb2 or Syb2 mutation/truncations was 2 μM.

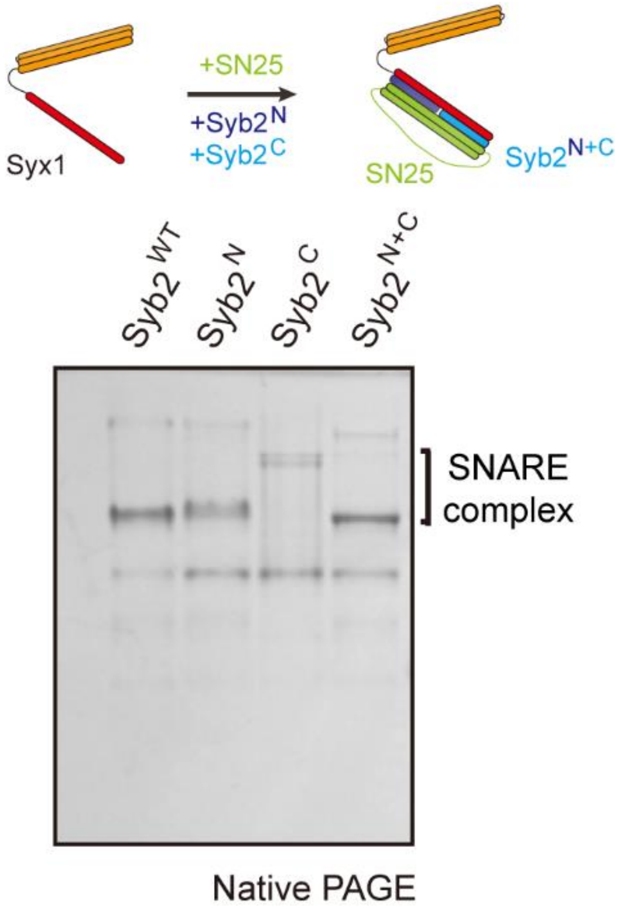

Supplementary Figure 3. Syb^{N}, Syb^{C}, and Syb2^{N+C} preserve the ability to form a ternary complex with Syx1 and SN25. Assembly of Syb2^{N}, Syb^{C} and Syb2^{N+C} with SN25 and Syx1 was assessed by native PAGE experiments. In the experiment, 4 μM Syx1, 5 μM SN25, 10 μM Syb2^{WT} or Syb2^{N} (residues 29–59, layers -7 to 0) and/or 10 μM Syb2^{C} (residues 60–96, layers +1 to +8) were mixed at 30 ℃ for 1 hours. The representative gel displayed is from one of three replicates. The scheme is illustrated at the top of the chart.

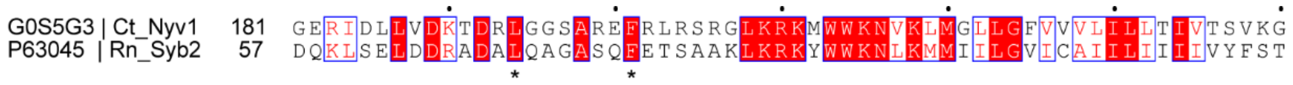

Supplementary Figure 4. Sequence alignment of Syb2 and Nyv1. Sequence alignment was performed by using Clustal Omega, and illustration of the result was achieved by using ESPript 3.0. Asterisks indicate the corresponding residues L194 and F201 of Nyv1 and L70 and F77 of Syb2. 100% consensus residues are shaded in red, similar residues are colored in red. Uniprot entries are indicated on the left of the sequences. Ct, *Chaetomium thermophilum*; Rn, *Rattus norvegicus*.

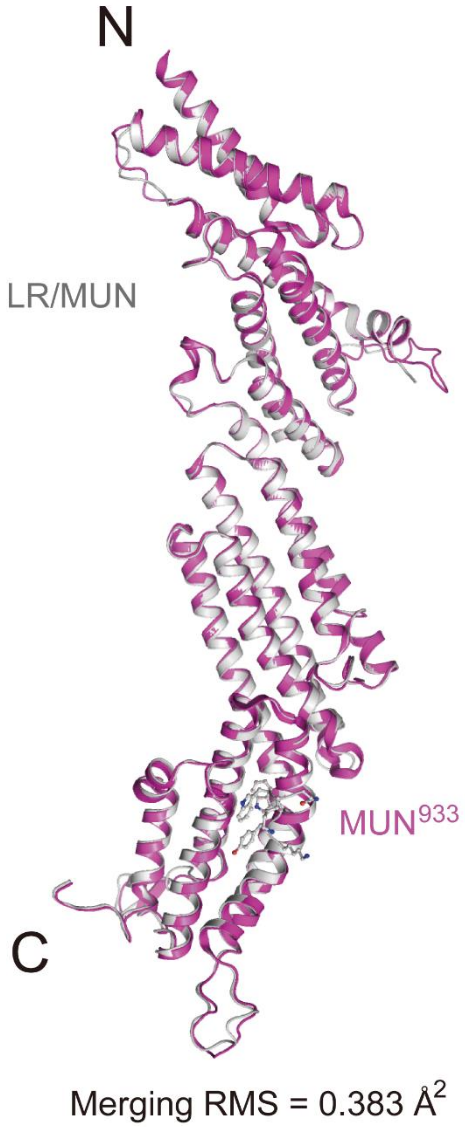

Supplementary Figure 5. The global structure of the MUN domain is unaffected when bound to Syb2. Superimposition of the crystal structure of Syb2 (LR)/MUN complex (grey) and the crystal structure of MUN^{933} (PDB entry: 4y21) (magenta) is shown. Superimposition process was performed using PyMol, and the merging root-mean-square (RMS) was 0.383 Å, as displayed in the diagram.

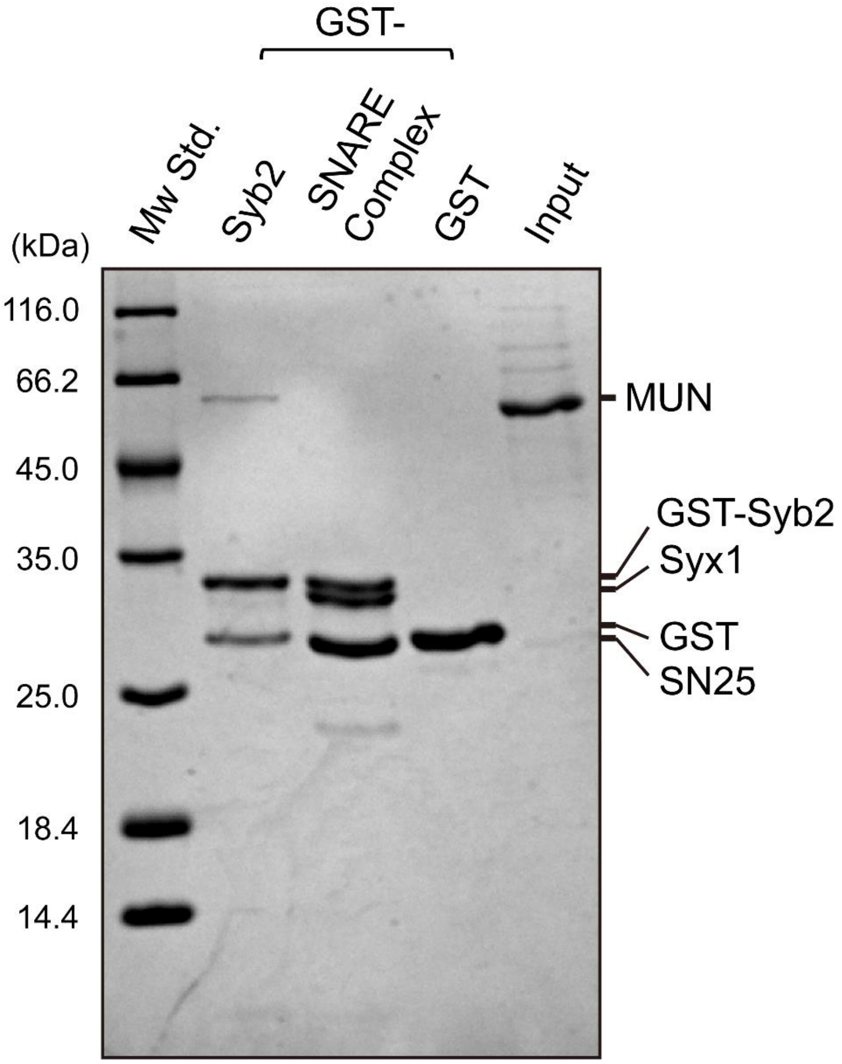

Supplementary Figure 6. The MUN domain prefers to bind isolated Syb2 than the *cis*-SNARE complex. Binding of the MUN domain to isolate Syb2 and to the *cis*-SNARE complex was measured by GST pull-down experiments. Purified GST-Syb2 (residues 29–96) was mixed with SN25 and Syx1 (residues 1–261) at 4℃ overnight to achieve full SNARE complex formation; 2 μM GST-Syb2 or GST-SNARE complex, 3 μM MUN were mixed with 10 μl 50% (v/v) glutathione Sepharose 4B affinity media (GE Healthcare) to a final volume of 50 μl at 4℃ for 3 hours. After washing out of the unbound proteins 3 times, the samples were mixed with 2 x SDS loading buffer, boiled for 10 minutes, and analyzed by SDS-PAGE. The representative gel displayed is from one of three replicates.

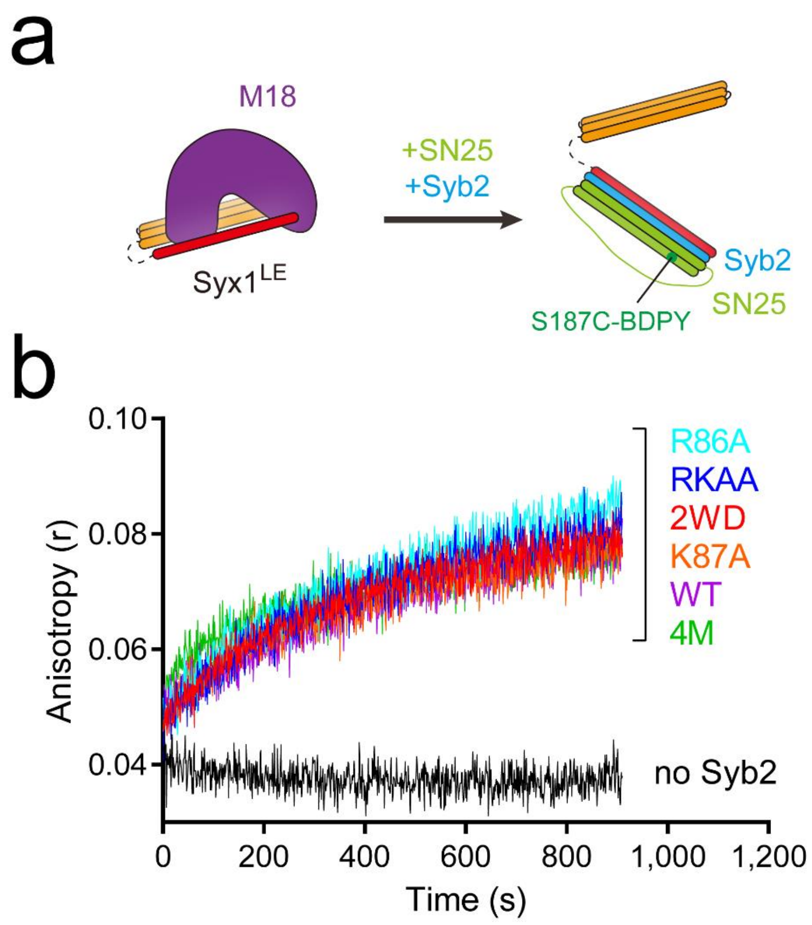

Supplementary Figure 7. The mutations in the Syb2 linker region do not interfere with the transition from the Munc18-1/Syx1^{LE} complex to the SNARE complex.

a Schematic diagram of MUN-independent transition from the Munc18-1/Syx1^{LE} complex to the SNARE complex. Fluorescent dye (BOPY) was labeled at residue S187C of SN25. The LE mutation (L165A/E166A) that destabilizes the folded structure of the Syx1 linker region between H_{abc} and H_3 domain was applied. **b** Analysis of the mutations in the Syb2 linker region in the transition of the Munc18-1/Syx1^{LE} complex to the SNARE complex by fluorescence anisotropy. In the experiments, co-expressed Munc18-1/Syx1^{LE} complex (5 μM) was mixed with 10 μM Syb2 (residues 29–96) or its mutants and 0.2 μM BDPY-labeled SN25; fluorescence anisotropies were acquired at 30 ℃.

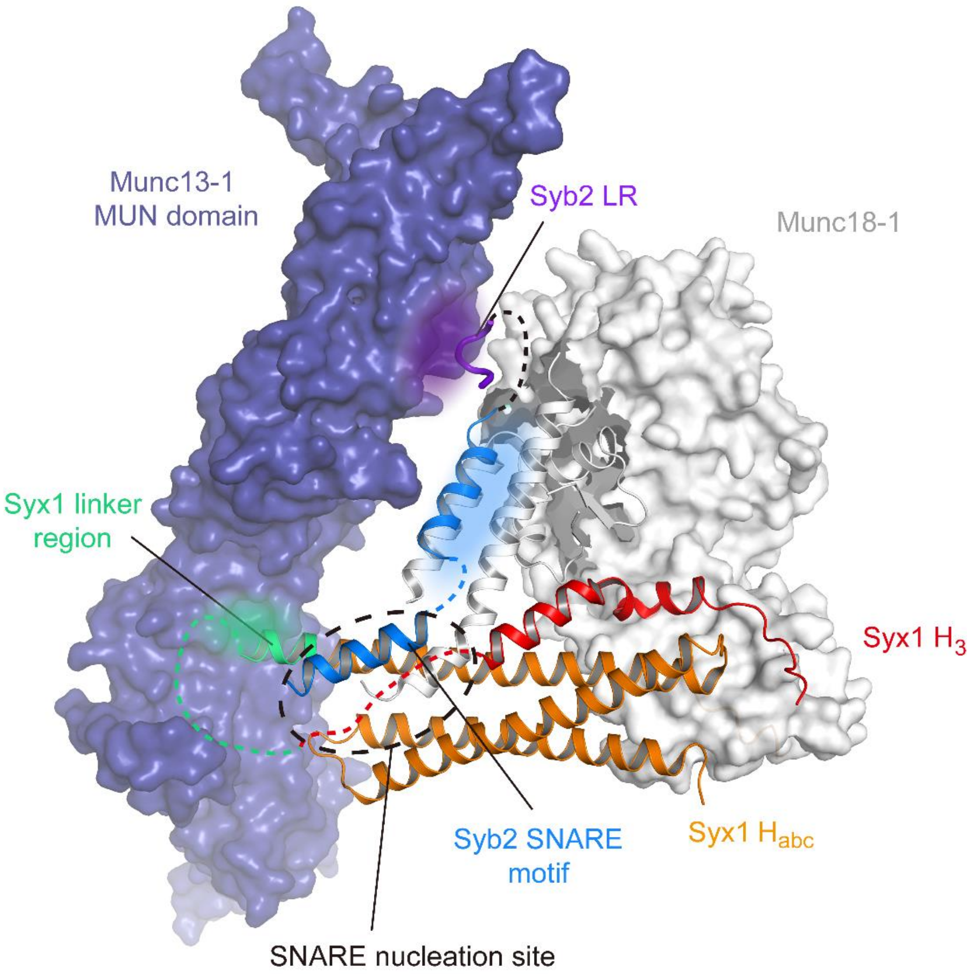

Supplementary Figure 8. Illustration of a quadruple MUN/Syb2/Munc18-1/Syx1 complex. The main architecture is based on the crystal structure of the Munc18-1/Syx1 complex (PDB entry: 3C98) and the Syb2/MUN complex. The "extended" conformation of Munc18-1 domain 3 (displayed as grey cartoons) is modeled based on the Munc18-1/Syx4 N-peptide complex (PDB entry: 3PUJ). The Syb2 SNARE motif (shown as blue cartoons) bound to Munc18-1 is modeled based on Vps16/Vps33/Nyv1 complex (PDB entry: 5BV0). Three determinate interaction interfaces, including i) "NF" pocket of the MUN domain and RI sequence of Syx1 linker region; ii) Syb2 LR and MUN (D1358); and iii) the Syb2 SNARE motif and Munc18-1 domain 3, are indicated by cyan green, purple, and blue shadows, respectively. The putative destabilized Syx1 linker region and the N-terminal H3 domain are shown as cyan green and red dashed lines, respectively. The Syx1 H_{abc} domain is shown as orange cartoons.

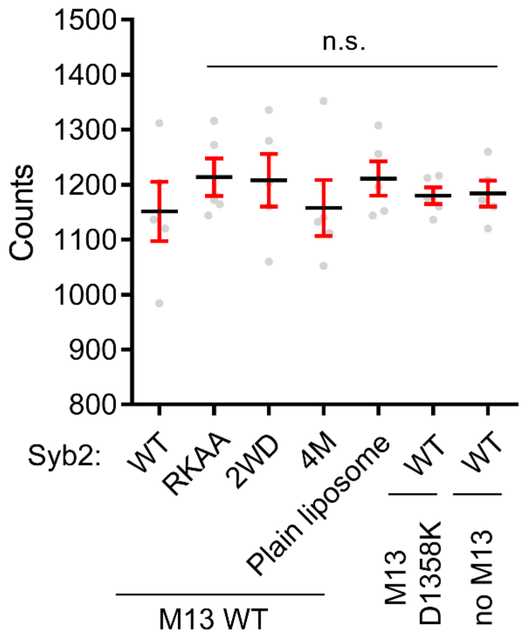

Supplementary Figure 9. Counting of the immobilized densities of the PM-vesicles. Quantification of the immobilized PM-vesicles in seven different channels are shown: Syb2^{WT}-SV-vesicles/wild-type M13, Syb2^{RKAA}-SV-vesicles/wild-type M13, Syb2^{2WD}-SV-vesicles/wild-type M13, Syb2^{4M}-SV-vesicles/wild-type M13, plain SV-vesicles/wild-type M13, Syb2^{WT}-SV-vesicles/M13-D1358K mutant, Syb2^{WT}-SV-vesicles only. Data are presented as the means ± SEM, with dots showing individual single-vesicle counts from 5 randomly chosen frames (n = 5). n.s., not significant, two-tailed t-test. Source data are provided as a Source Data file.

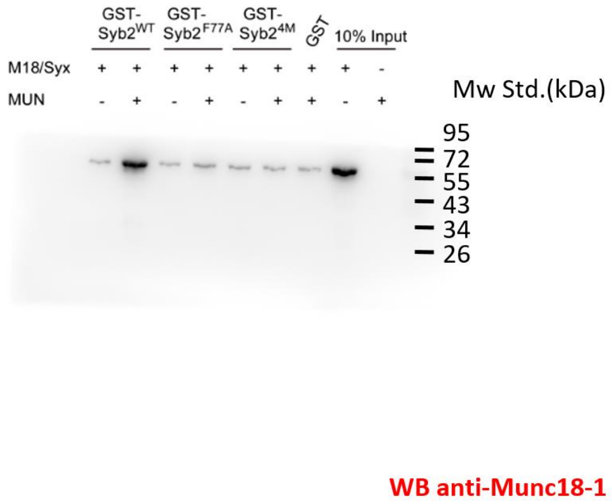

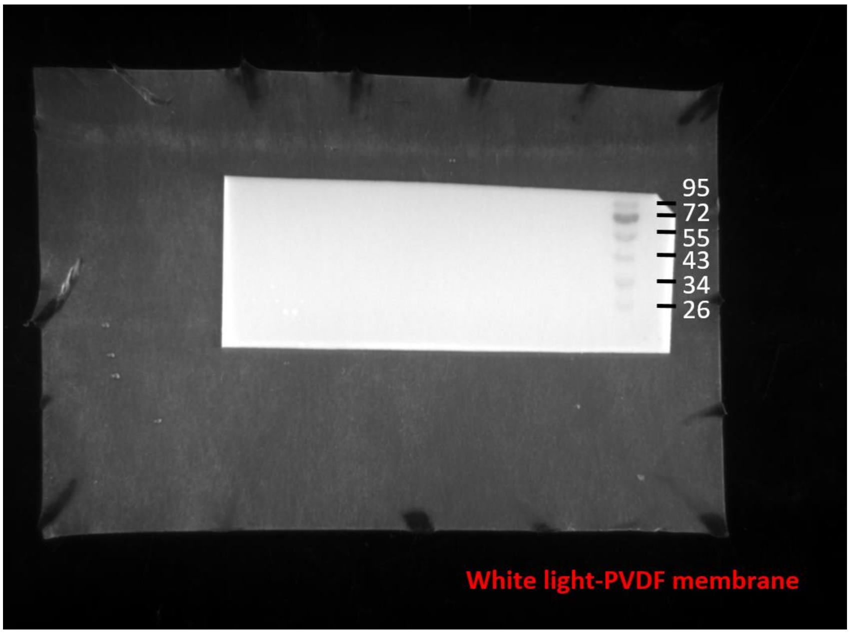

Supplementary Figure 10. Uncropped scan of western blots in Figure. 6c. Upper panel displays uncropped chemical luminescence as shown in Figure 6c; lower panel displays the whole PVDF membrane under white light containing pre-stained molecular weight standard as indicated.

Supplementary Table 1. Summary table of mutations or truncations used in the study

Name	Derived from	Vector	Residue no.	Mutations
Syb2^{-7}	Syb2	pGEX-KG	29–96	L32A
$\text{Syb2}^{\Delta-7}$	Syb2	pGEX-KG	35–96	
$\text{Syb2}^{\Delta-6}$	Syb2	pGEX-KG	39–96	
$\text{Syb2}^{\Delta+8}$	Syb2	pGEX-KG	29–83	
$\text{Syb2}^{\Delta+7}$	Syb2	pGEX-KG	29–80	
$\text{Syb2}^{\Delta+6}$	Syb2	pGEX-KG	29–76	
$\text{Syb2}^{\Delta+5}$	Syb2	pGEX-KG	29–73	
$\text{Syb2}^{\Delta+4}$	Syb2	pGEX-KG	29–69	
Syb2^{N}	Syb2	pGEX-KG	29–59	
Syb2^{C}	Syb2	pGEX-KG	60–96	
$\text{Syb2}^{49\text{-}96}$	Syb2	pGEX-KG	49–96	S61C
Syb2^{RKAA}	Syb2	pGEX-KG	29–96	R86A, K87A
Syb2^{2WD}	Syb2	pGEX-KG	29–96	W89D, W90D
Syb2^{4M}	Syb2	pGEX-KG	29–96	R86A, K87A, W89D, W90D
Syb2^{K83A}	Syb2	pGEX-KG	29–96	K83A
Syb2^{K85A}	Syb2	pGEX-KG	29–96	K85A
Syb2^{R86A}	Syb2	pGEX-KG	29–96	K86A
Syb2^{K87A}	Syb2	pGEX-KG	29–96	K87A
Syb2^{L70A}	Syb2	pGEX-KG	29–96	L70A
Syb2^{F77A}	Syb2	pGEX-KG	29–96	F77A
Syb2^{G73P}	Syb2	pGEX-KG	29–96	G73P
Full-length Syb2^{RKAA}	Syb2	pGEX-KG	1–116	R86A, K87A
Full-length Syb2^{2WD}	Syb2	pGEX-KG	1–116	W89D, W90D

Full-length Syb2^{4M}	Syb2	pGEX-KG	1–116	R86A, K87A, W89D, W90D
SN25$^{\Delta+7}$	SNAP-25	pET28a	1–198	Four native Cys to Ser
SN25$^{\Delta+5}$	SNAP-25	pET28a	1–191	Four native Cys to Ser
SN25$^{\Delta+2}$	SNAP-25	pET28a	1–180	Four native Cys to Ser
SN25$^{\Delta+1}$	SNAP-25	pET28a	1–177	Four native Cys to Ser
SN25$^{\Delta0}$	SNAP-25	pET28a	1–173	Four native Cys to Ser
SN25$^{\Delta-1}$	SNAP-25	pET28a	1–170	Four native Cys to Ser
SN1	SNAP-25	pGEX-KG	1–83	
Munc18-1/Syx1^{LE}	Munc18-1/Syx1	pETDuet-1	1–594 (Munc18-1) 1–261 (Syx1)	L165A, E166A (Syx1)
MUN^{D1358K}	Munc13-1 MUN domain	pGEX-KG	933–1407, EF, 1453–1531	D1358K
MUN^{Q1362K}	Munc13-1 MUN domain	pGEX-KG	933–1407, EF, 1453–1531	Q1362K
MUN^{D1366K}	Munc13-1 MUN domain	pGEX-KG	933–1407, EF, 1453–1531	D1366K
C_1C_2BMUN^{D1358K}	Munc13-1 C_1C_2BMUN fragment	pFastBac HtB	529–1407, EF, 1453–1531	D1358K
