# Supplementary material for: Munc18 and Munc13 serve as a functional template to orchestrate neuronal SNARE complex assembly
Source: Nat Commun. 2019 Jan 8;10:69. doi: 10.1038/s41467-018-08028-6 (PMC6325239; doi:10.1038/s41467-018-08028-6)
Supplement: Supplementary file 4 — Reporting Summary [file 41467_2018_8028_MOESM4_ESM.pdf]

nature research

Corresponding author(s): Cong Ma

Reporting Summary

Nature Research wishes to improve the reproducibility of the work that we publish. This form provides structure for consistency and transparency in reporting. For further information on Nature Research policies, see Authors & Referees and the Editorial Policy Checklist.

Statistical parameters

When statistical analyses are reported, confirm that the following items are present in the relevant location (e.g. figure legend, table legend, main text, or Methods section).

n/a	Confirmed	
☐	☒	The exact sample size (n) for each experimental group/condition, given as a discrete number and unit of measurement
☐	☒	An indication of whether measurements were taken from distinct samples or whether the same sample was measured repeatedly
☐	☒	The statistical test(s) used AND whether they are one- or two-sided<br>*Only common tests should be described solely by name; describe more complex techniques in the Methods section.*
☒	☐	A description of all covariates tested
☒	☐	A description of any assumptions or corrections, such as tests of normality and adjustment for multiple comparisons
☐	☒	A full description of the statistics including central tendency (e.g. means) or other basic estimates (e.g. regression coefficient) AND variation (e.g. standard deviation) or associated estimates of uncertainty (e.g. confidence intervals)
☐	☒	For null hypothesis testing, the test statistic (e.g. F, t, r) with confidence intervals, effect sizes, degrees of freedom and P value noted<br>*Give P values as exact values whenever suitable.*
☒	☐	For Bayesian analysis, information on the choice of priors and Markov chain Monte Carlo settings
☒	☐	For hierarchical and complex designs, identification of the appropriate level for tests and full reporting of outcomes
☒	☐	Estimates of effect sizes (e.g. Cohen's d, Pearson's r), indicating how they were calculated
☐	☒	Clearly defined error bars<br>*State explicitly what error bars represent (e.g. SD, SE, CI)*

Our web collection on statistics for biologists may be useful.

Software and code

Policy information about availability of computer code

Data collection	NIS Elements 4.3.1, Felix GX 4.2.2 were used for data collection
Data analysis	NIS Elements 4.3.1, Igor Pro 7.0.8.1, Image J 1.50g, MATLAB 7.11.0, Prism 6.0.1, Pymol 1.5.0.3 were used for data analysis

For manuscripts utilizing custom algorithms or software that are central to the research but not yet described in published literature, software must be made available to editors/reviewers upon request. We strongly encourage code deposition in a community repository (e.g. GitHub). See the Nature Research guidelines for submitting code & software for further information.

Data

Policy information about availability of data

All manuscripts must include a data availability statement. This statement should provide the following information, where applicable:

- Accession codes, unique identifiers, or web links for publicly available datasets
- A list of figures that have associated raw data
- A description of any restrictions on data availability

Data for the structure reported here have been deposited in the PDB under the accession code 6A30, all other data supporting the findings of this study are available within the paper and its supplementary information files.

Field-specific reporting

Please select the best fit for your research. If you are not sure, read the appropriate sections before making your selection.

☒ Life sciences ☐ Behavioural & social sciences ☐ Ecological, evolutionary & environmental sciences

For a reference copy of the document with all sections, see nature.com/authors/policies/ReportingSummary-flat.pdf

Life sciences study design

All studies must disclose on these points even when the disclosure is negative.

Sample size	For in vitro experiments, sample size was at least 3; for in vivo experiments, sample size was ranged from 12 to 28, with 3 or 4 independent cultures. Statistical hypothesis using two-tailed t-test assumes that the samples are normal distribution
Data exclusions	No data were excluded from all analyses
Replication	At least 3 independent replications were applied to each experiment in the manuscript
Randomization	Samples allocation was random
Blinding	All investigators were blinded to group allocation during data collection and analysis

Reporting for specific materials, systems and methods

Materials & experimental systems

n/a	Involved in the study	
☒	☐	Unique biological materials
☐	☒	Antibodies
☐	☒	Eukaryotic cell lines
☒	☐	Palaeontology
☐	☒	Animals and other organisms
☒	☐	Human research participants

Methods

n/a	Involved in the study	
☒	☐	ChIP-seq
☒	☐	Flow cytometry
☒	☐	MRI-based neuroimaging

Antibodies

Antibodies used	Munc18-1 rabbit polyclonal antibody, Proteintech #11459-1-AP;<br>Syntaxin-1a mouse monoclonal antibody, Proteintech #66437-1-lg.
Validation	The antibodies are validated for WB in human tissues, human cell lines, rat and mouse cell lines as reported by the manufacturer. Data provided in the manuscript validated the antibody for WB with bacterial expressed recombinant protein (origins: rat).

Eukaryotic cell lines

Policy information about cell lines

Cell line source(s)	HEK293T cells (CRL-11268) were from ATCC, dissociated cortex neurons were obtained from newborn pups
Authentication	Each cell line used in the study was authenticated
Mycoplasma contamination	All cell lines are negative for mycoplasma contamination
Commonly misidentified lines<br>(See ICLAC register)	None

Animals and other organisms

Policy information about studies involving animals; ARRIVE guidelines recommended for reporting animal research

Laboratory animals	New born wild-type Rattus norvegicus with random sex were used in the study
Wild animals	The study did not involve wild animals
Field-collected samples	The study did not involve samples collected from the field
